# Comparison of the Effectiveness and Safety of Chlorthalidone and Hydrochlorothiazide in Patients With Hypertension: A Meta-Analysis

**DOI:** 10.7759/cureus.38184

**Published:** 2023-04-27

**Authors:** Raja Ram Khenhrani, Ijeoma Nnodebe, Anurag Rawat, Rahul Adwani, Ammara Ghaffar, Sapna Devi, Muhammad Sohaib Afzal, Muhammad Usama

**Affiliations:** 1 Internal Medicine, Liaquat University of Medical and Health Sciences, Karachi, PAK; 2 Medicine, Basingstoke and North Hampshire Hospital, Basingstoke, GBR; 3 Interventional Cardiology, Himalayan Institute of Medical Sciences, Dehradun, IND; 4 Medicine, Dow University of Health Sciences, Karachi, PAK; 5 Neurology, King Edward Medical University, Lahore, PAK; 6 Internal Medicine, Medical College, Liaquat University of Medical and Health Sciences, Karachi, PAK; 7 Medicine, Louisiana State University Health Sciences Center, Shreveport, USA; 8 Neurology, Sheikh Zayed Medical College & Hospital, Rahim Yar Khan, PAK

**Keywords:** meta-analysis, blood pressure, hypertension, chlorthalidone, hydrochlorothiazide

## Abstract

The aim of this study was to compare the effectiveness and safety of chlorthalidone and hydrochlorothiazide in patients with hypertension. The present meta-analysis was reported according to the Preferred Reporting Items for Systematic Reviews and Meta-Analyses (PRISMA) guidelines. Our search for relevant articles was conducted on PubMed, Scopus, and CINAHIL databases from their inception until March 31, 2023. Keywords used to search for relevant articles included "hydrochlorothiazide," "chlortalidone," "hypertension," "cardiovascular," and "blood pressure." The outcomes assessed in this meta-analysis included changes in systolic blood pressure (SBP) and diastolic blood pressure (DBP). Myocardial infarction, stroke, and all-cause mortality were also assessed. For safety analysis, we evaluated the risk of hypokalemia between the two groups. Any disagreement between the two authors in the data extraction process was resolved through discussion. Eight studies fulfilled the inclusion criteria included in the present meta-analysis. Our analysis showed that chlorthalidone was superior to hydrochlorothiazide in controlling both SBP and DBP, with no significant heterogeneity reported. However, there was no significant difference between the two groups in terms of the risk of myocardial infarction, stroke, all-cause mortality, and hospitalization due to heart failure. The hypokalemia rate was reported to be higher with chlorthalidone compared to hydrochlorothiazide.

## Introduction and background

Hypertension is a syndrome that involves multiple neuroendocrine, hemodynamic, and metabolic abnormalities [1]. The prevalence of hypertension has been rising worldwide [2]. Hypertension is associated with an increased risk of complications and mortality from cardiovascular illnesses [3]. For over 40 years, thiazide diuretics have been the primary medication for the management of hypertension in most patients. Two of these medications are chlorthalidone, which is considered a thiazide-like agent, and hydrochlorothiazide, which is considered a thiazide-type agent [4]. The U.S. Food and Drug Administration approved both drugs more than 50 years ago [5]. Recent observational studies have shown that the two drugs decreased the risk of cardiovascular events at a similar rate [6]. However, the risk of adverse events, including hypokalemia, is highest in patients receiving chlorthalidone. Thiazide-type diuretics, such as hydrochlorothiazide at a daily dose of 12.5-25 mg, have traditionally been the first choice for treating most patients with high blood pressure (BP). However, in recent times, chlorthalidone has become popular as a replacement for hydrochlorothiazide due to its superior clinical outcomes and effectiveness in lowering BP [7].

Medicare spending revealed that in 2020, around 1.5 million individuals were prescribed chlorthalidone, whereas 11.5 million were prescribed hydrochlorothiazide, despite guidelines recommending chlorthalidone as the preferred option [8]. The reason for this difference between recommended and actual use may be linked to the perception that chlorthalidone carries a higher risk of adverse effects such as electrolyte imbalances, even though there is no clear evidence to suggest differences in cardiovascular outcomes [6].

Even though chlorthalidone and hydrochlorothiazide are structurally similar compounds, they are quite different pharmacokinetically. Hydrochlorothiazide is different from chlorthalidone in that the latter has a long half-life and a large distribution volume because of its extensive partitioning into red blood cells [9]. A half dose of chlorthalidone is more effective in reducing systolic blood pressure (SBP) compared to hydrochlorothiazide, primarily due to its BP-lowering efficacy throughout the nighttime hours [10]. Thus, it is hypothesized that the differences in the persistence of BP-lowering effectiveness would play an important role in differences in arterial stiffness and central BPs [10].

Despite being similar in structure and mechanism of action, some studies suggest that chlorthalidone may be more effective in reducing BP and preventing cardiovascular events than hydrochlorothiazide. However, the evidence is conflicting, and the safety profiles of the two drugs have not been fully explored. Therefore, a comprehensive meta-analysis comparing the effectiveness and safety of chlorthalidone and hydrochlorothiazide in patients with hypertension is warranted. The aim of the present meta-analysis was to compare the effectiveness and safety of chlorthalidone and hydrochlorothiazide in patients with hypertension.

## Review

Meta-analysis

The present meta-analysis was reported according to the Preferred Reporting Items for Systematic Reviews and Meta-Analyses (PRISMA) guidelines.

Search Strategy and Study Selection

Our search for relevant articles was conducted on PubMed, Scopus, and CINAHIL databases using keywords such as "hydrochlorothiazide," "chlortalidone," "hypertension," "cardiovascular," and "blood pressure" from their inception until March 31, 2023. All identified articles were imported into EndNote X9, and duplicates were removed. The initial screening of titles and abstracts was followed by a full-text screening based on predefined inclusion and exclusion criteria. Additionally, we manually searched the reference lists of all included studies.

Inclusion and Exclusion Criteria

For inclusion in this meta-analysis, studies had to meet the following criteria: (1) observational studies and randomized controlled trials (RCTs) that assessed different doses of hydrochlorothiazide and chlorthalidone and (2) studies that evaluated hydrochlorothiazide and chlorthalidone alone or in combination with other antihypertensive drugs. We excluded case reports, case series, and editorials, as well as studies published in languages other than English. There were no restrictions placed on the year of publication.

Data Extraction and Outcome Measures

Data from the included studies were extracted using a pre-designed Microsoft Excel Spreadsheet. One author extracted the data, and the second author cross-checked the data and entered it into the RevMan software for analysis. The data extracted included the author's name, year of publication, study design, sample size, dose, and follow-up period. The outcomes assessed in this meta-analysis included changes in SBP and diastolic blood pressure (DBP) in mmHg. Myocardial infarction, stroke, and all-cause mortality were also assessed. For safety analysis, we evaluated the risk of hypokalemia between the two groups. Any disagreement between the two authors in the data extraction process was resolved through discussion.

Statistical Analysis

We used RevMan Version 5.4.1 (The Cochrane Collaboration, London, United Kingdom) to perform the data analysis. We calculated the mean difference (MD) along with the 95% confidence interval (CI) to compare the effect of drugs on continuous outcome variables. For categorical outcomes, we calculated the hazard ratio (HR) with a 95% CI. A p-value of less than 0.05 was considered significant. To measure the variation in treatment effects between the studies, we applied the Cochran Q and the I-square statistics. A p-value of less than 0.10 indicated the presence of heterogeneity, and an I-square value greater than 50% indicated significant heterogeneity.

Results

**Figure 1:**
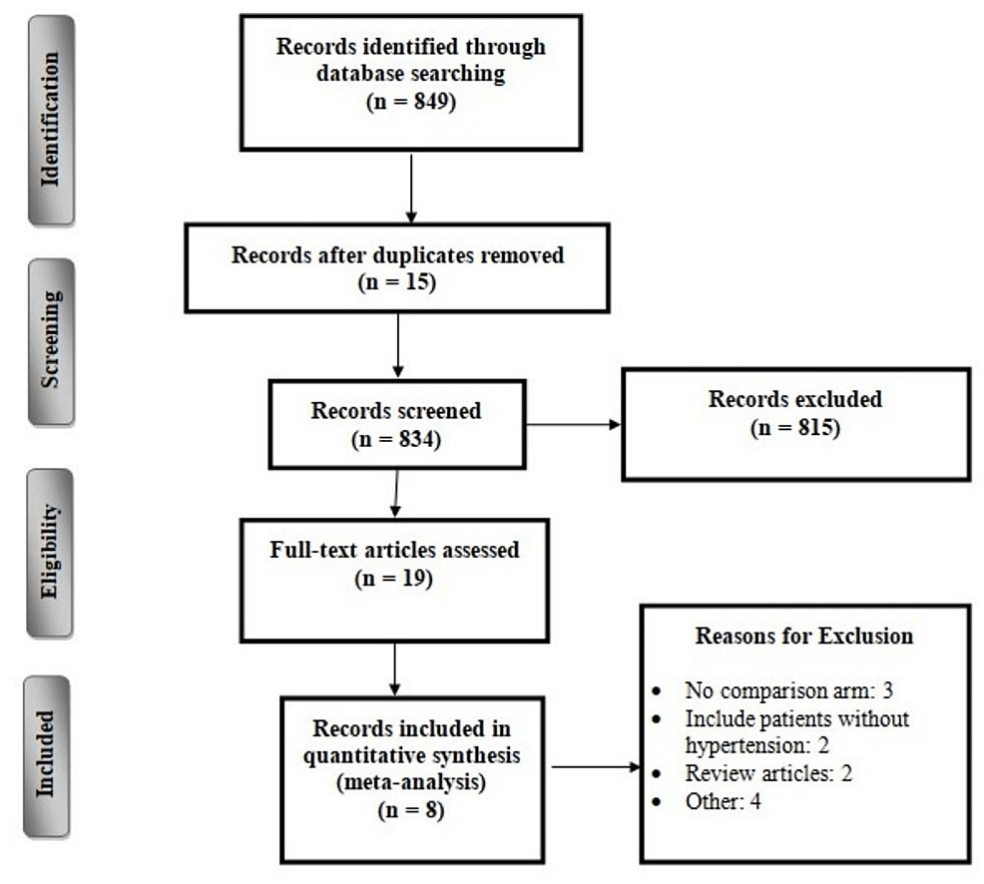
PRISMA flowchart of selection of studies PRISMA, Preferred Reporting Items for Systematic Reviews and Meta-Analyses

Figure 1 shows the study selection process. Online database search yielded 849 articles. After removing duplicates, 834 articles were initially screened using titles and abstracts. Among them, 815 studies were excluded. Full text of 19 studies was obtained, and detailed assessment of eligibility criteria was conducted. In the end, eight studies fulfilled the inclusion criteria and were included in the present meta-analysis. Summarized extracted data regarding the author name, publication year, sample size and follow-up period are presented in Table 1. The duration of follow-up was between 2 and 60 months. Four studies were RCTs.

**Table 1:** Characteristics of included studies RCT, randomized controlled trial

Author	Year	Study design	Groups	Dose	Sample size	Follow-up	Age (years)	Males (%)
Bakris et al. [11]	2012	RCT	Chlorthalidone	12.5 to 25 mg	295	2.5 months	56.8 vs 55.9	52.1 vs 50.7
			Hydrochlorothiazide	12.5 to 25 mg	292			
Dhalla et al. [12]	2013	Retrospective cohort	Chlorthalidone	12.5 to 50 mg	10384	60 months	73 vs 73	40.7 vs 41
			Hydrochlorothiazide	12.5 to 50 mg	19489			
Ernst et al. [10]	2006	Cross-over	Chlorthalidone	12.5 to 25 mg	14	2 months	46 vs 49	50 vs 56
			Hydrochlorothiazide	25 to 50 mg	16			
Hripcsak et al. [13]	2020	Retrospective cohort	Chlorthalidone	12.5 mg	36859	>12 months	49 vs 48.2	48.2 vs 38.9
			Hydrochlorothiazide	25 mg	692371			
Ishani et al. [14]	2022	RCT	Chlorthalidone	12.5 to 25 mg	6756	30 months	72.4 vs 72.5	96.7 vs 96.9
			Hydrochlorothiazide	25 to 50 mg	6767			
Kwon et al. [15]	2013	Cross-over	Chlorthalidone	12.5 mg	28	2 months	NR	NR
			Hydrochlorothiazide	25 mg	28			
Pareek et al. [16]	2009	RCT	Chlorthalidone	6.25 to 12.5 mg	66	3 months	48.9 vs 51.9	45.5 vs 46.2
			Hydrochlorothiazide	12.5 to 25 mg	65			
Pareek et al. [17]	2016	RCT	Chlorthalidone	6.25 mg	16	4 months	41.1 vs 46.8	56.2 vs 50
			Hydrochlorothiazide	12.5 mg	20			

Change in SBP and DBP

**Figure 2:**
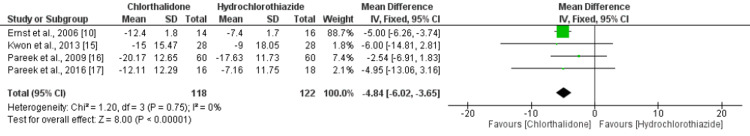
Forest plot comparing the effect of chlorthalidone and hydrochlorothiazide on systolic blood pressure Sources: [10,15-17]

**Figure 3:**
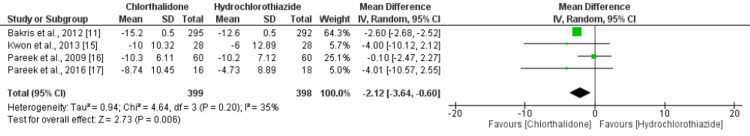
Forest plot comparing the effect of chlorthalidone and hydrochlorothiazide on diastolic blood pressure Sources: [11,15-17]

Four studies compared the change in SBP from baseline. The mean reduction in SBP was significantly greater in patients receiving chlorthalidone compared to patients receiving hydrochlorothiazide (MD: -4.84, 95% CI: -6.02 to -3.65), as shown in Figure 2. No heterogeneity was reported among the study results (I-square=0%, p-value=0.75). A pooled analysis of four studies assessing the change in DBP from baseline showed that the mean reduction of DBP was significantly greater in patients receiving chlorthalidone compared to patients receiving hydrochlorothiazide (MD: -2.12, 95% CI: -3.64 to -0.60), as shown in Figure 3. No significant heterogeneity was reported among the study results (I-square=35%, p-value=0.20).

Myocardial Infarction and Stroke

**Figure 4:**
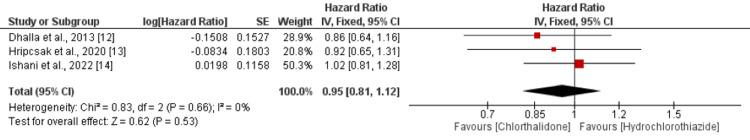
Forest plot comparing the effect of chlorthalidone and hydrochlorothiazide on myocardial infarction Sources: [12-14]

**Figure 5:**
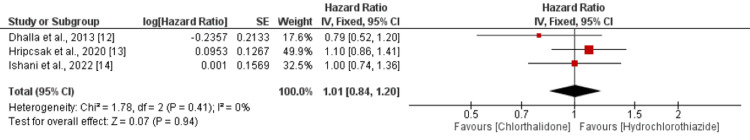
Forest plot comparing the effect of chlorthalidone and hydrochlorothiazide on stroke Sources: [12-14]

Three studies were conducted to compare the risk of myocardial infarction. The results showed no significant difference between the two groups in terms of the risk of myocardial infarction (HR: 0.95, 95% CI: 0.81, 1.12), as shown in Figure 4. No significant heterogeneity was reported among the study results (I-square=0%, p-value=0.66). Similarly, three studies were conducted to compare the risk of stroke. The results showed no significant difference between the patients who received chlorthalidone and those who received hydrochlorothiazide in terms of the risk of stroke (HR: 1.01, 95% CI: 0.84, 1.20), as shown in Figure 5. No significant heterogeneity was reported among the study results (I-square=0%, p-value=0.41).

All-Cause Mortality and Hospitalization due to Heart Failure

**Figure 6:**
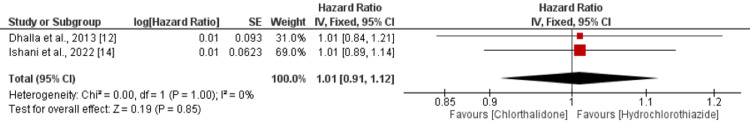
Forest plot comparing the effect of chlorthalidone and hydrochlorothiazide on all-cause mortality Sources: [12,14]

**Figure 7:**
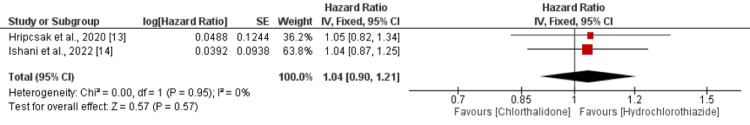
Forest plot comparing the effect of chlorthalidone and hydrochlorothiazide on hospitalization due to heart failure Sources: [13-14]

Two studies were conducted to compare the risk of all-cause mortality between patients who received chlorthalidone and those who received hydrochlorothiazide. The results showed no significant difference between the two groups in terms of the risk of all-cause mortality (HR: 1.01, 95% CI: 0.91-1.12), as shown in Figure 6. No significant heterogeneity was reported among the study results (I-square=0%, p-value=1.00). Furthermore, no significant difference was reported between the two groups in terms of the risk of hospitalization due to heart failure (HR: 1.04, 95% CI: 0.90-1.21), as shown in Figure 7.

Hypokalemia

**Figure 8:**
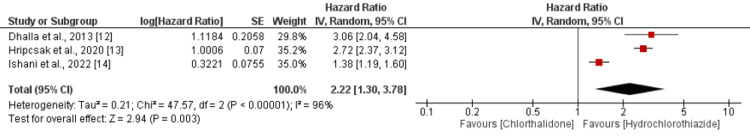
Forest plot comparing the effect of chlorthalidone and hydrochlorothiazide on hypokalemia Sources: [12-14]

The forest plot of the hypokalemia is shown in Figure 8. The hazard of hypokalemia in patients receiving chlorthalidone was significantly higher compared to patients receiving hydrochlorothiazide (HR: 2.22, 95% CI: 1.30-3.78). High heterogeneity was reported among the study results (I-square=96%).

Discussion

The aim of our meta-analysis was to compare the efficacy, effectiveness, and safety of chlorthalidone and hydrochlorothiazide as monotherapy or in combination with other antihypertensive treatments in individuals with hypertension. The meta-analysis reported superiority of chlorthalidone in terms of control of DBP and SBP compared to hydrochlorothiazide. However, no significant differences were reported between the two groups in regard to the risk of myocardial infarction, stroke, all-cause mortality, and hospitalization due to heart failure. In terms of hypokalemia, the risk was reported to be higher with chlorthalidone compared to hydrochlorothiazide.

All over the world, hydrochlorothiazide is used more often than chlorthalidone [18]. However, in recent years, it has been actively debated whether chlorthalidone and hydrochlorothiazide need to be considered interchangeable agents. Evidence indicates that chlorthalidone may be a more favorable option than hydrochlorothiazide [19]. Two studies that indirectly compared hydrochlorothiazide and chlorthalidone using network meta-analysis had contrasting results. Psaty et al. included three clinical trials that used chlorthalidone and three that used other low-dose diuretics [20]. They assessed mortality and cardiovascular endpoints and reported no significant difference between chlorthalidone and other thiazide-like drugs. On the other hand, a study conducted by Roush et al. [21], which included six clinical trials, reported that chlorthalidone is likely to be more effective compared to hydrochlorothiazide. Our meta-analysis is different from these two analyses as we included recently conducted trials and studies with direct comparison between hydrochlorothiazide and chlorthalidone.

Chlorthalidone has effective BP control compared to hydrochlorothiazide due to its unique pharmacokinetic profile, which allows it to stay active for a longer period, possibly due to its wider distribution in the body, including the red blood cells [9,22]. This sustained effect is particularly effective during the night and early morning and is thought to be responsible for chlorthalidone's known advantages in reducing cardiovascular disease and death rates [23]. The use of thiazides can lower the chances of cardiovascular problems, but there is a debate on which type of thiazide to use. Earlier research has indicated that chlorthalidone is more effective than hydrochlorothiazide in preventing cardiovascular problems. It seems that chlorthalidone has a longer-lasting impact, leads to better control of BP throughout the day, and has other beneficial effects [10-11]. However, multiple observational studies suggest different effects on cardiovascular outcomes, ranging from no impact to an increased risk of cardiovascular issues with chlorthalidone compared to hydrochlorothiazide [12-13].

The present meta-analysis reported a higher risk of hypokalemia in patients receiving chlorthalidone compared to hydrochlorothiazide. All three studies that assessed this outcome reported a higher risk of hypokalemia with chlorthalidone [12-14]. However, only one study was an RCT. A study conducted by Lund and Ernst [24] did not specifically report a significant difference in the incidence of hypokalemia or hyponatremia between hydrochlorothiazide and chlorthalidone, but they reported that patients who received chlorthalidone were more likely to discontinue treatment compared to patients who received hydrochlorothiazide. There are several reasons why patients treated with chlorthalidone may discontinue treatment, but one of the possible reasons is that it can cause electrolyte imbalances. Chlorthalidone is a stronger and longer-lasting antihypertensive medication than hydrochlorothiazide, which is the most commonly prescribed medication, but it can also lower serum potassium levels more significantly [25].

The current meta-analysis showed that chlorthalidone is better at controlling BP compared to hydrochlorothiazide. However, only one of the included studies reported a significant difference in SBP and DBP between the two groups [10]. Therefore, in the future, well-designed, large randomized trials are necessary to compare the effectiveness and safety of chlorthalidone and hydrochlorothiazide and to reach conclusive evidence that can help healthcare professionals make decisions about the prescription of medications for patients with hypertension. In the practice setting, medications need to be prescribed to help patients reach patient-relevant BP goals while considering the risk of electrolyte imbalance [26].

Study Limitations

The present meta-analysis has certain limitations. Firstly, only four RCTs were included in this meta-analysis. Out of four trials, only one trial assessed long-term outcomes and included myocardial infarction and stroke. Due to the limited number of studies, we were unable to perform subgroup analysis. More RCTs with larger sample sizes are needed to confirm our findings and identify specific patient populations that may benefit from chlorthalidone or hydrochlorothiazide.

## Conclusions

Our analysis showed that chlorthalidone was superior to hydrochlorothiazide in controlling both SBP and DBP, with no significant heterogeneity reported. However, there was no significant difference between the two groups in terms of the risk of myocardial infarction, stroke, all-cause mortality, and hospitalization due to heart failure. The risk of hypokalemia was reported to be higher with chlorthalidone. Further studies are needed to confirm these findings and to investigate the long-term effects of chlorthalidone and hydrochlorothiazide in patients with hypertension. When it comes to actual medical practice, physicians should prescribe medications that aid patients in achieving their BP targets, taking into account the possibility of electrolyte imbalance as a potential risk factor. It is crucial for healthcare providers to balance the benefits and risks of medication and its adverse events before prescribing it to ensure that patients receive the best possible treatment with minimal negative outcomes.

## References

[1] Weber MA, Laragh JH (1993). Hypertension: steps forward and steps backward: the Joint National Committee Fifth Report. Arch Intern Med.

[2] Mills KT, Bundy JD, Kelly TN (2016). Global disparities of hypertension prevalence and control: a systematic analysis of population-based studies from 90 countries. Circulation.

[3] Flint AC, Conell C, Ren X (2019). Effect of systolic and diastolic blood pressure on cardiovascular outcomes. N Engl J Med.

[4] Ernst ME, Moser M (2009). Use of diuretics in patients with hypertension. N Engl J Med.

[5] Cranston WI, Juel-Jensen BE, Semmence AM, Jones RP, Forbes JA, Mutch LM (1963). Effects of oral diuretics on raised arterial pressure. Lancet.

[6] Krause T, Lovibond K, Caulfield M, McCormack T, Williams B (2011). Management of hypertension: summary of NICE guidance. BMJ.

[7] Edwards C, Hundemer GL, Petrcich W (2021). Comparison of clinical outcomes and safety associated with chlorthalidone vs hydrochlorothiazide in older adults with varying levels of kidney function. JAMA Netw Open.

[8] DiPette DJ, Goughnour K, Zuniga E (2020). Standardized treatment to improve hypertension control in primary health care: The HEARTS in the Americas Initiative. J Clin Hypertens (Greenwich).

[9] Sica DA (2006). Chlorthalidone: has it always been the best thiazide-type diuretic?. Hypertension.

[10] Ernst ME, Carter BL, Goerdt CJ, Steffensmeier JJ, Phillips BB, Zimmerman MB, Bergus GR (2006). Comparative antihypertensive effects of hydrochlorothiazide and chlorthalidone on ambulatory and office blood pressure. Hypertension.

[11] Bakris GL, Sica D, White WB (2012). Antihypertensive efficacy of hydrochlorothiazide vs chlorthalidone combined with azilsartan medoxomil. Am J Med.

[12] Dhalla IA, Gomes T, Yao Z (2013). Chlorthalidone versus hydrochlorothiazide for the treatment of hypertension in older adults: a population-based cohort study. Ann Intern Med.

[13] Hripcsak G, Suchard MA, Shea S (2020). Comparison of cardiovascular and safety outcomes of chlorthalidone vs hydrochlorothiazide to treat hypertension. JAMA Intern Med.

[14] Ishani A, Cushman WC, Leatherman SM (2022). Chlorthalidone vs. hydrochlorothiazide for hypertension-cardiovascular events. N Engl J Med.

[15] Kwon BJ, Jang SW, Choi KY (2013). Comparison of the efficacy between hydrochlorothiazide and chlorthalidone on central aortic pressure when added on to candesartan in treatment-naïve patients of hypertension. Hypertens Res.

[16] Pareek A, Basavanagowdappa H, Zawar S, Kumar A, Chandurkar N (2009). A randomized, comparative study evaluating the efficacy and tolerability of losartan-low dose chlorthalidone (6.25 mg) combination with losartan-hydrochlorothiazide (12.5 mg) combination in Indian patients with mild-to-moderate essential hypertension. Expert Opin Pharmacother.

[17] Pareek AK, Messerli FH, Chandurkar NB (2016). Efficacy of low-dose chlorthalidone and hydrochlorothiazide as assessed by 24-h ambulatory blood pressure monitoring. J Am Coll Cardiol.

[18] Cooney D, Milfred-LaForest S, Rahman M (2015). Diuretics for hypertension: hydrochlorothiazide or chlorthalidone?. Cleve Clin J Med.

[19] Dorsch MP, Gillespie BW, Erickson SR, Bleske BE, Weder AB (2011). Chlorthalidone reduces cardiovascular events compared with hydrochlorothiazide: a retrospective cohort analysis. Hypertension.

[20] Psaty BM, Lumley T, Furberg CD (2004). Meta-analysis of health outcomes of chlorthalidone-based vs nonchlorthalidone-based low-dose diuretic therapies. JAMA.

[21] Roush GC, Holford TR, Guddati AK (2012). Chlorthalidone compared with hydrochlorothiazide in reducing cardiovascular events: systematic review and network meta-analyses. Hypertension.

[22] Sica DA (2009). Chlorthalidone - a renaissance in use?. Expert Opin Pharmacother.

[23] National Heart, Lung and Blood Institute, NIH NIH (2023). National Heart, Lung and Blood Institute, NIH. Systolic Blood Pressure Intervention Trial (SPRINT) Overview. https://www.nhlbi.nih.gov/science/systolic-blood-pressure-intervention-trial-sprint-study.

[24] Lund BC, Ernst ME (2012). The comparative effectiveness of hydrochlorothiazide and chlorthalidone in an observational cohort of veterans. J Clin Hypertens (Greenwich).

[25] Peterzan MA, Hardy R, Chaturvedi N, Hughes AD (2012). Meta-analysis of dose-response relationships for hydrochlorothiazide, chlorthalidone, and bendroflumethiazide on blood pressure, serum potassium, and urate. Hypertension.

[26] Chobanian AV (2008). Does it matter how hypertension is controlled?. N Engl J Med.

